# Remote ischaemic conditioning in the context of type 2 diabetes and neuropathy: the case for repeat application as a novel therapy for lower extremity ulceration

**DOI:** 10.1186/s12933-016-0444-z

**Published:** 2016-09-09

**Authors:** J. A. Epps, N. A. Smart

**Affiliations:** School of Science and Technology, The University of New England, Armidale, NSW 2351 Australia

**Keywords:** Diabetes, Neuropathy, Diabetic foot, Ulcer, Remote ischaemic conditioning, Repeat remote ischaemic conditioning, Endothelial function, Neovascularisation, Endothelial progenitor cells

## Abstract

An emerging treatment modality for reducing damage caused by ischaemia–reperfusion injury is ischaemic conditioning. This technique induces short periods of ischaemia that have been found to protect against a more significant ischaemic insult. Remote ischaemic conditioning (RIC) can be administered more conveniently and safely, by inflation of a pneumatic blood pressure cuff to a suprasystolic pressure on a limb. Protection is then transferred to a remote organ via humoral and neural pathways. The diabetic state is particularly vulnerable to ischaemia–reperfusion injury, and ischaemia is a significant cause of many diabetic complications, including the diabetic foot. Despite this, studies utilising ischaemic conditioning and RIC in type 2 diabetes have often been disappointing. A newer strategy, repeat RIC, involves the repeated application of short periods of limb ischaemia over days or weeks. It has been demonstrated that this improves endothelial function, skin microcirculation, and modulates the systemic inflammatory response. Repeat RIC was recently shown to be beneficial for healing in lower extremity diabetic ulcers. This article summarises the mechanisms of RIC, and the impact that type 2 diabetes may have upon these, with the role of neural mechanisms in the context of diabetic neuropathy a focus. Repeat RIC may show more promise than RIC in type 2 diabetes, and its potential mechanisms and applications will also be explored. Considering the high costs, rates of chronicity and serious complications resulting from diabetic lower extremity ulceration, repeat RIC has the potential to be an effective novel advanced therapy for this condition.

## Introduction

Diabetes-related foot disease is a frequent complication of type 2 diabetes mellitus (T2DM), with up to 25 % of diabetic patients eventually developing foot ulceration [1–3]. The physical, emotional and financial costs for patients, their carers and the community are substantial. The utilisation of health care services and associated expenses are high in diabetic patients with ulcers [4]. In the US, it has been shown that the costs of foot ulceration caused by diabetes and related complications amount to between $9 and $13 billion per annum using conservative measures [1, 5]. Responsible for a large proportion of hospital admissions and health care costs in diabetes [2], such figures are likely to increase. The prevalence of T2DM is escalating worldwide [2, 6]. Although international data are scarce, an improved survival rate of diabetic patients has been reported in wealthier nations in particular [7, 8]. A potential consequence of this may be a higher prevalence of diabetic ulcers, especially as duration of diabetes is a major risk factor for the development of lower extremity ulceration [9].

Arguably the most feared complication of diabetic foot ulceration is the need for amputation. A UK study observed this occurred in 19 % of cases over a 5 year period in patients with a diabetic ulcer present for 2 weeks [10]. In a 2016 systematic review, amputation rates associated with diabetic ulceration ranged from 5 to 35 % [11]. Rates have improved in some higher income countries by 40 to 60 % [12], however data from countries of lower income and poorer access to health care (yet a higher prevalence of diabetes) are notably lacking [8, 12]. Further, some population groups even within wealthy nations are clearly at much higher risk of amputation [13–15]. In Australia, the Indigenous diabetic community is at particularly high risk. A 2000–2008 analysis revealed in some regions this population experienced up to 27 times higher risk of minor amputation and 38 times higher risk of major amputation [13]. The benefits of improved access to primary health foot care, in addition to multidisciplinary foot clinics, cannot be overemphasised. They are highlighted by the impressive 72 % reduction, following adjustment of variables, in lower extremity amputation rates identified in a 15 year longitudinal observational study of a West Australian city [16]. Despite this, from 2002 to 2012 there was a 30 % overall increase in diabetes-related amputations in well-resourced Australia [17], with one of the highest rates of amputation amongst developed nations [18]. The need to improve statistics worldwide for diabetic ulcer prevalence, complication rate and cost is undeniable, and new therapeutic options must be considered. This article will assess the potential for repeat remote ischaemic conditioning (RRIC) as a novel adjuvant treatment in diabetic lower extremity ulceration.

## Methods

Utilising PubMed and Scopus databases in January 2016, publications relating to all forms of local and remote ischaemic conditioning were searched. Articles with an emphasis on neural mechanisms, diabetes or comorbidity in ischaemic conditioning, RIC or RRIC were identified in particular. The papers retrieved were examined, and further relevant references obtained by reviewing cited articles and cross-referencing. Alerts utilising Scopus and PubMed were established in these search areas over a 7 month period ending August 2016, enabling access to the most current research. Further searches were performed as required for other material covered in this article, including diabetic lower extremity ulceration, diabetic neuropathy and diabetic mobilopathy.

## Pathogenesis of lower extremity ulceration in diabetes

Sustained hyperglycaemia and associated abnormal metabolic pathways in T2DM lead to peripheral neuropathy, micro- and macro- vascular peripheral arterial disease, mechanical changes and subsequent foot trauma (e.g. Charcot neuroarthropathy), high plantar pressure, increased susceptibility to infection, skin changes resulting from autonomic neuropathy, increased pro-inflammatory cytokines, decreased neovascularisation and tissue regeneration, decreased neuropeptides involved in angiogenesis, and an altered extracellular matrix [2, 10, 19–22]. Diabetic neuropathy and bone marrow microangiopathy have been shown to prevent mobilisation of haematopoietic stem cells (stem cell “mobilopathy”), crucial in the response to ischaemia [23, 24], and can result in abnormal neurovascular regulation [25]. Decreased production of stromal cell-derived factor-1α in diabetic wounds impairs homing of endothelial progenitor cells (EPCs) to the ulcer region [26]. Diabetes also impairs the release of vascular endothelial growth factor (VEGF), normally upregulated in response to hypoxia, with resultant abnormal neovascularisation [27, 28]. All of these factors may contribute to the development of lower extremity ulceration or prevent healing. Poor foot self-care, age, and the presence of other microvascular complications are also important risk factors [29].

## Treatment of diabetic lower extremity ulcers

The standard principles of management of diabetic ulcers, ideally co-ordinated by a multidisciplinary team, are debridement (utilising various methods), infection control, pressure offloading, optimising glycaemic control, application of dressings and surgical intervention (revascularisation or orthopaedic), addressed in recent comprehensive reviews of these treatments [2, 22, 30–34]. Newer advanced or potential adjunctive therapies include hyperbaric oxygen therapy [2, 22, 30–34], bioengineered skin [2, 22, 30–32, 34], negative pressure wound therapy [2, 22, 30–34], growth factors [2, 22, 30–34], electrophysical therapy [2, 22, 30–32], and stem cells [22, 30, 33]. NorLeu3-angiotensin (1–7), substance P and extracellular matrix proteins have also been studied [reviewed in 22]. Many of these newer therapies are either expensive, difficult to implement, lack evidence for substantial benefit, or lack consensus on which method of administration is most effective for each therapy [31]. Even though extensive research has been undertaken investigating a wide range of treatment options and optimal management, chronic ulcers are still all too frequent. The average ulcer takes approximately 3 months to heal, and at least a half of diabetic ulcers recur within 3 years [3]. Further research into treatment options is therefore needed.

## Potential treatment candidate: ischaemic conditioning

### Background

It is clear there is a great need to identify novel treatments to improve diabetic ulcer healing. One potential candidate is ischaemic conditioning. This phenomenon was first discovered in 1986 by Murry, Jennings and Reimer, when a study in dogs showed 4 cycles consisting of 5 min of complete occlusion of a coronary artery, followed by 5 min of reperfusion, decreased the size of a subsequent induced myocardial infarction by up to 75 % when compared to control [35]. Termed ischaemic *pre*conditioning, this intervention protects against myocardial ischaemia–reperfusion injury [36]. Ischaemic *post*conditioning was discovered in 2003, when it was identified that the ischaemic stimulus remained cardioprotective even when applied after myocardial reperfusion [37]. In 1993, it was found that administering cycles of preconditioning to a coronary artery supplying a different region of myocardium to the site of a subsequent infarction remained cardioprotective [38]. An extension of this concept lead to a study in 2002, which demonstrated that applying the ischaemic preconditioning more conveniently to skeletal muscle also provided cardioprotection [39]. Termed remote ischaemic conditioning (RIC), this has been categorised into several forms: remote ischaemic preconditioning (RIPC), remote ischaemic perconditioning and remote ischaemic postconditioning [40]. Yet further subcategories exist when the stimulus is applied up to 24 h before or greater than 15 min after the target organ ischaemia: delayed remote ischaemic pre- and post-conditioning respectively [40]. Although a variety of organs have been utilised as the source of the ischaemic stimulus in RIC studies [36], skeletal muscle in upper or lower limbs have predominantly been used.

Ischaemic preconditioning has been shown to provide both an early and late window of protection. The first phase lasts for 2–3 h and commences immediately after the significant ischaemic episode. The second phase commences between 12 and 24 h after the episode and persists for approximately 3 days of protection [41, 42].

RIC has recently been studied when applied repeatedly (RRIC). There has been considerable variation in the timeframe of administration, ranging from one bout of 5 cycles of 5 min of ischaemia and reperfusion applied once or twice daily [43], to three 5 min cycles applied once per fortnight [44]. The period of intervention has ranged from 1 week of application of RRIC [45] to 300 days of RRIC [43]. In RIC and RRIC interventions, either single or bilateral limbs have been studied, and upper or lower limbs, as the effector organ(s). Different systolic pressures have been used in RIC to induce limb ischaemia; typically a pneumatic blood pressure cuff is inflated to 200 mmHg. The optimal protocols for all forms of RIC and RRIC are not yet known, and need to be standardised [46, 47].

Despite disappointing results in some translational studies in humans [48], mounting evidence points towards RIC as a potentially powerful new therapeutic tool for an increasingly wider range of conditions. Early studies focused upon cardiac [reviewed in 36] and renal [reviewed in 49, 50] endpoints. More recently, the use of RIC has been studied in subarachnoid haemorrhage management, thromboembolic stroke, cardiovascular complications following major vascular surgery, cutaneous blood flow, transplantation surgery [51, 52], skin and lung applications [51], skeletal muscle [53], endothelial function [54, 55] (particularly in RRIC) [reviewed in 56, 57], hypertension [58], and exercise performance [46], some showing more promise than others.

## Remote ischaemic conditioning mechanisms

### Neural mechanisms

The mechanisms of RIC are complex and have been the focus of intense study. The exact mechanisms remain unknown, but it is evident that both neural and humoral pathways are involved and that the two interact [36, 40]. It is likely that the ischaemic stimulus causes afferent C fibre sensory nerves to be activated by local release of autacoids, such as calcitonin gene related peptide (CGRP), bradykinin and adenosine [40, 59–63]. Separate animal model studies have demonstrated that the cardioprotective effects of RIPC are lost if the sensory nerve to the ischaemic limb [64, 65], spinal cord [66, 67], the dorsal motor nucleus of the vagus nerve [68], the vagus nerve [67, 69], renal nerves [70, 71], or the posterior gastric branch of the vagus nerve [72], are transected or silenced. These key studies reveal how crucial neural pathways are, from sensory afferent fibres to the efferent autonomic reflexes, in transmitting the signal for cardioprotection in RIPC. Importantly, it was found by Basalay et al. [73] that vagotomy or sectioning nerves of the ischaemic limb abolished RIPC cardioprotective effects, although not the effects of remote ischaemic *post*conditioning. This study demonstrated that both these forms of remote ischaemic conditioning are cardioprotective, but utilise very different pathways. The implications in the context of the diabetic patient will be discussed below, but it is apparent that humoral pathways are more prominent in remote ischaemic postconditioning.

### Humoral mechanisms

Despite the importance of neural pathways, overwhelming evidence also exists for a humoral mechanism in RIC. This was demonstrated effectively in a study by Shimizu et al. where protection was transferred humorally between animals and even between species, by transfusion of dialysate of plasma, from a preconditioned human to a rabbit that received no RIC stimulus. The cardioprotection was also not reduced when transferred to a denervated rabbit heart [74]. A substantial amount of research has been undertaken to try and determine the humoral effector(s) of RIC, of which many have been studied, and is beyond the scope of this article. Readers are directed to two very detailed recent review articles [36, 75] for comprehensive analysis and discussion of the latest evidence. To summarise, the most significant or likely humoral effector candidates resulting in a reduced myocardial infarct size (the most frequently used endpoint for RIC studies [48]) would appear to be adenosine, CGRP, bradykinin, nitric oxide (NO) and plasma nitrite, hypoxia inducible factor 1-α, erythropoietin, stromal-derived factor-1α, and microRNAs [reviewed in 36, 40]. Apolipotrotein A1 [76, 77] and kynurenic acid [78] are also potential effectors. Extracellular vesicles, which can transport microRNAs, are likely to be important in conveying the cardioprotective signal [79]. Evidence exists for the involvement of numerous substances, but none appear to act as an effector in isolation and it is likely that many pathways are involved [36]. The late phase of RIC appears to include nitric oxide synthase, heat shock proteins and also cyclooxygenase-2 [reviewed in 52, 80]. Most investigations of the late phase of ischaemic conditioning have assessed the mechanism of local (not remote) ischaemic preconditioning [81]. It is felt the mechanisms are similar, but that both the early and late phase of RIPC are reliant on neural pathways to a greater degree than local ischaemic preconditioning [42]. The late phase of RIPC has also been shown to result in the mobilisation of bone marrow-derived stem cells, including CD34+ cells, into both the peripheral blood and damaged myocardium, which have the capacity to induce angiogenesis and endothelial repair [82, 83].

### Neural and humoral mechanisms are closely linked

Shimizu et al.’s rabbit study proving cardioprotection could be transferred to a denervated heart [74] is often cited as evidence that neural pathways are less crucial to the mechanism of RIC. This had appeared the case for the target organ effects, but certainly does not exclude neural pathways as being essential for the generation of the protective signal, nor for the release of humoral factor(s) either locally or via neural pathways to other non-target organs [47]. In a very recent study by Pickard et al.’s research team, an ex vivo rat heart received dialysate from donor rats following RIC administration to the donor. Cardioprotection from a subsequent induced myocardial infarction was abrogated by administration of either atropine or hexamethonium to the recipient, immediately before dialysate treatment [69]. Vagotomy prior to the RIC protocol in donor rats also abrogated the RIC cardioprotection in the naïve recipient rat heart [69]. These findings are evidence that both neural and humoral pathways are integrally linked, at least in the early phase of RIPC for cardioprotection, and also confirm that neural pathways are important as far downstream as the target organ itself. Pickard et al. suspect the likely involvement of intrinsic cardiac ganglia in the mechanism [69]. In addition to neural and humoral pathways, there is evidence that an anti-inflammatory effect is produced by RIC through alteration of inflammatory gene expression [84], neutrophil function [85], and interleukin-10 upregulation [86].

### End-organ protection

The end result of cardioprotection occurs via activation of the reperfusion injury salvage kinase (RISK) pathways, protein kinase C pathways (via adenosine triphosphate-sensitive potassium channels) and survival activating factor enhancement (SAFE) during reperfusion. Mitochondrial influx of molecules and cell death during reperfusion is then prevented by closure of the mitochondrial permeability transitions pore [36, 46, 48, 52, 75]. The mechanism of neuroprotection, in contrast, is less well studied as an end point of RIC than cardioprotection. It is likely to involve similar mitochondrial pro-survival pathways preventing apoptosis, the neuroprotective activation of synaptic *N*-methyl-d-aspartate receptors and increased cerebral blood flow [87, 88]. The mechanism of renoprotection in RIC is even less well understood and researched, although cell cycle arrest is thought to be involved [89].

## Nociceptive-induced conditioning

In a study by Jones et al. [90] using a mouse model, and in a study by Gross et al. [91] in dogs, the intriguing phenomenon of remote preconditioning of trauma was identified. The animals in both studies were subjected to a shallow wound being administered prior to inducing myocardial infarction, and this procedure was found to be cardioprotective, reducing infarct sizes by 80 and 59 % respectively. This protection could be blocked by administration of local anaesthesia at the wound site [90]. The application of topical capsaicin also had a similar effect in decreasing myocardial infarction size [90, 92]. These findings have been termed nociceptive-induced remote conditioning [66]. Redington et al. took the further step to demonstrate that electroacupuncture also was cardioprotective, with many similarities to RIPC and previous nociceptive conditioning studies [93].

Considering the impressive findings of the nociceptive-inducing cardioprotection studies, we propose that this may also be a substantial confounding factor in many translational RIPC studies, resulting in misleading conclusions. An example would be human studies in RIPC used prior to coronary artery bypass graft (CABG) surgery. The control groups do not receive the RIPC protocol in these trials, but both groups necessarily require a large central sternotomy wound. Applying the conclusions from the nociceptive-induced remote myocardial conditioning studies described above, one could argue that the central sternotomy wound in itself could provide conditioning, to which RIPC may not contribute significant additional benefit. The impact, location and timing of any co-administered local, regional and/or general anaesthesia could also potentially alter nociceptive-induced conditioning, varying with the protocol used. The same concepts may apply to any medical or surgical procedure or treatment involving trauma or nociception. Logically, it could therefore be anticipated that study groups receiving non-invasive treatment for relatively painless conditions would benefit most from RIC, with no co-conditioning from pain or trauma. The promising early use of RIC for ischaemic stroke is one such example [87].

To ignore the potential for nociceptive co-conditioning in a study may lead to incorrect conclusions regarding the response to RIPC. We suspect it would be important to evaluate previous and future study designs carefully, to establish whether pain or trauma could be a confounding factor due to co-conditioning. To the authors’ knowledge, the lowest threshold or dose necessary to induce nociceptive conditioning has not yet been established. If superficial skin wounds [91] and electroacupuncture [93] are sufficient, it remains to be seen whether other very minor procedures, such as intramuscular injections, cannulations and capillary blood sampling, can trigger nociceptive conditioning. Should small, minor stimuli be sufficient, it is plausible that the earliest recorded therapeutic use of nociceptive conditioning in humans was in fact 100BC (or earlier) in China, when acupuncture practice was first clearly documented [94].

## Difficulties in clinical translation of remote ischaemic conditioning

Clinical studies that show benefits, such as the application of RIC in CABG surgery and elective and primary percutaneous coronary intervention (PCI), have not always been reproducible [36, 48, 95–99]. The most frequent positive findings have been seen in the use of RIC intervention in primary PCI [47]. Results from animal RIC studies are typically more promising than those in humans [98, 100]. Conflicting findings may be due to differences between the animal models studied and humans [48, 98, 100], or to limitations in study designs [48]. In human studies, it is also very likely to be due to co-morbidity [48, 98, 101], co-medication and differing anaesthetic protocols [98, 101–105], heterogeneity in study designs [48, 62], and other factors that cannot be predicted due to an incomplete understanding of the mechanisms and interaction of neural and humoral pathways in RIC. As a consequence, confounding factors are likely to be unwittingly present in studies, which could result in misleading conclusions.

## Difficulties in clinical translation of ischaemic conditioning in diabetes

As ischaemia plays an important role in the pathophysiology of many complications of T2DM, and the diabetic state appears particularly vulnerable to ischaemia–reperfusion injury [106], it seems intuitive that patients with T2DM would benefit from ischaemic conditioning. However, mixed and neutral findings have been reported in preclinical and clinical studies in diabetes [48, 107–110], with particularly poor results in preclinical postconditioning studies [48]. The majority of human studies in diabetes have assessed cardioprotection in acute myocardial infarction (AMI) following ischaemic conditioning, with disappointing results [98]. Conversely, evidence for the preservation of RIPC cardioprotection in human diabetics undergoing percutaneous coronary intervention was found in a 2014 meta-analysis [111].

The number of human ischaemic conditioning studies specifically in T2DM is, however, very small, particularly with the gold-standard endpoint of myocardial infarction size, and most of these do not assess *remote* ischaemic conditioning efficacy [98, 100, 107]. The first early window of protection in diabetes has also received considerably more study than the second window. In studies performed with favourable cardioprotective outcomes for ischaemic (pre- and post-) conditioning in diabetes, a common finding is that an increased ischaemic stimulus is often required [112–115].

### Potential factors in type 2 diabetes limiting efficacy

#### Cardiomyocyte changes

Complex changes have been detected in diabetic hearts, in human and animal model studies (particularly assessing postconditioning [48]), that are likely to result in the myocardium being less responsive to ischaemic conditioning and RIC. These include impaired kinase and signalling pathways. Specifically, these are altered extracellular signal-regulated kinase (ERK) 1/2 [116, 117], decreased phosphorylation of glycogen synthase kinase-3 beta [117, 118], impaired [112, 113, 117] or chronic [114] Akt phosphorylation and Akt activity [119], effects on mitochondrial adenosine triphosphate (ATP)-dependent potassium channels [106, 120–124], decreased CGRP [125], reduced adenosine [108], altered bioavailability of NO [126] and abnormalities of other apoptotic pathways [127, 128]. In a human RIPC diabetes study, raised *O*-linked β-*N*-acetylglucosamine was found to be associated with a state of chronic cardioprotection, with subsequent RIPC providing no additional benefit [129]. Perplexingly, the diabetic state has also been found to limit ischaemia–reperfusion injury [110, 130]. This was highlighted in a study by Ma et al. using streptozotocin-induced diabetic rat models, although protection was limited to 2 weeks following induction of diabetes. Akt activation, increased NO and VEGF formation leading to angiogenesis were identified as likely mechanisms [131]. A study by Ravingerová et al. using a similar diabetic rat model also concluded that early diabetes conferred resistance to myocardial necrosis [132]. In this instance, RIC may offer little additional benefit.

#### Hyperglycaemia

Hyperglycaemia and resultant oxidative stress are likely to be involved in the changes of the abovementioned pathways in diabetes [108, 133]. Whilst many studies have demonstrated the loss of cardioprotection from ischaemic conditioning in hyperglycaemia [116, 121, 134–137], this loss of cardioprotection was identified in a Zucker obese rat study *before* the onset of hyperglycaemia [123]. This demonstrated that hyperglycaemia is not the only underlying mechanism in the loss of cardioprotection in type 2 diabetes. To further complicate the picture, studies of hyperglycaemia in animal models of type 1 diabetes demonstrated that acute diabetes is cardioprotective, but these benefits were lost after 6 weeks of onset of diabetes [reviewed in 109]. In addition to hyperglycaemia, insulin is also an important factor in cardioprotection. Activation of Akt by administration of insulin has an inhibitory effect on ischaemic preconditioning efficacy [138]. The restoration of cardioprotection from ischaemic postconditioning with return to normoglycaemia, through transplantation of islet cells, has been identified in a type 1 diabetic mouse model study [116]. In a separate rat study, however, insulin pretreatment had very different findings, with no restoration of cardioprotective effects from ischaemic postconditioning [139].

Most research assessing the sensitivity to ischaemia–reperfusion injury has focused upon type 1 rather than type 2 diabetes [109]. Findings have often been conflicting, perhaps due to the differing methods of inducing diabetes, duration and severity of hyperglycaemia, animal models used, study protocols, and difficulties in comparing ex vivo, in vivo and in vitro scenarios [109].

#### Impaired endothelial progenitor cell mobilisation

Diabetes has a negative impact on EPC mobilisation in response to ischaemia [140], particularly in the context of diabetic neuropathy [141, 142]. It has been demonstrated that EPC mobilisation occurs following RIPC in both mice and humans [82, 83], and this appears likely to contribute significantly to the late phase cardioprotective effects of RIPC [82]. We therefore hypothesise that this may be another important and under-recognised mechanism behind the poorer response to RIPC in T2DM, particularly in the rarely-studied late phase of protection. Poor EPC mobilisation following AMI is known to be associated with adverse outcomes, especially in diabetes [143], and results in impaired neovascularisation [142]. Interestingly, diabetic mobilopathy has improved following administration of dipeptidyl peptidase-4 (DPP-4) inhibition [144].

#### Comorbidity and co-medication

Evidence exists that many conditions frequently occurring in T2DM also have a negative effect on cardioprotection in ischaemic conditioning. These include hyperlipidaemia, particularly in ischaemic postconditioning [98, 100, 145, 146], obesity [123, 147], and hypertension [100, 148]. Ventricular hypertrophy abolished cardioprotection in some animal model studies and not others [100, 149]. Chronic kidney disease surprisingly does not appear to abrogate the protective effects of ischaemic conditioning [98] and RIPC shows promise in providing renoprotection from acute injury [89], particularly contrast-induced acute kidney injury [50]. Diabetic neuropathy is known to abrogate the effect of RIPC [64], and is outlined in the section below.

Many medications used in the management of diabetes and its complications have been shown to have an effect upon ischaemic conditioning. Some are inherently cardioprotective, providing pharmacological conditioning. Examples include insulin [102, 150], metformin [151–153], sitagliptin [151], exenatide [154], sildenafil [102, 155], beta blockers [156] and potentially ACE inhibitors [102]. It is suspected that ischaemic conditioning may offer limited additional benefit when applied concurrently with pharmacological conditioning [95]. Other medication, such as statins [157], glimepiride [158, 159] and losartan or valsartan (angiotensin II receptor type 1 blockers) [160] have demonstrated augmentation or restoration of cardioprotection from ischaemic conditioning in diabetes and/or hyperglycaemia. Yet other classes abrogate the effects of ischaemic conditioning altogether, including older sulfonylureas such as glibenclamide [reviewed in 151]. Despite the knowledge about the effects of these medications, study findings of these effects are not always consistent, limiting further understanding. For example, in a retrospective analysis assessing the impact of likely confounders of RIC in CABG surgery outcomes, statins and cardioprotective antihypertensives were reported as not altering RIC cardioprotection [161].

It is essential to note that the majority of studies assessing the impact of comorbidity and co-medication upon cardioprotection in T2DM have used a local (not remote) ischaemic conditioning protocol [100], and small animal models [48, 151]. Many of the preclinical studies assessing the impact of medication used in human T2DM have been conducted utilising non-diabetic animals [151]. Most of the diabetic animal models and protocols used would not reflect the duration of T2DM and extent of diabetic complications seen in humans (including neuropathy), nor the true impact of polypharmacy, drug doses used in clinical practice, lifestyle choices and considerable comorbidity frequently encountered in T2DM [48, 100, 109]. Although such preclinical studies are much needed in T2DM, it is difficult to be certain of the degree of translatability and relevance through extrapolation of their key findings to human T2DM, particularly in the context of RIC. Thus, it is more than likely that these issues contribute to conflicting results from many ischaemic conditioning and RIC clinical studies in T2DM.

Readers are referred to comprehensive reviews of the impact of diabetes in ischaemic conditioning of the myocardium [98, 107, 108, 145], and medications commonly prescribed in T2DM [98, 151], for a more in-depth discussion of the difficulties in clinical translation of RIC in T2DM.

### Neural pathways in remote ischaemic conditioning and neuropathy of type 2 diabetes

Both the early and late phases of RIPC are dependent upon neural pathways, identified in a key human study by Loukogeorgakis et al. assessing the effects of RIPC on endothelial function as an endpoint [42]. More evidence for the requirement of a neural pathway in RIPC, specifically in the diabetic population, comes from another crucial study by Jensen and colleagues, who successfully demonstrated that intact peripheral nerves in human T2DM subjects were critical for generating the dialysable, transferrable cardioprotective factors that reduced myocardial infarct size in rabbits [64]. Jensen et al. found that the protective effect was the same in both non-diabetic subjects and diabetics without peripheral neuropathy, but that there was no protective effect in the dialysate obtained from diabetic patients with peripheral neuropathy, even with an intensified RIPC stimulus [64].

#### Peripheral neuropathy

Of interest is that Jensen et al.’s study protocol utilised the upper arm for the RIPC administration. The exact location of the peripheral neuropathy in the T2DM neuropathy group is not documented in the article [64]. Unless in advanced cases of peripheral sensorimotor neuropathy, sensory deficits occur predominantly in the lower limb neural pathways, as the neuropathy is nerve length-dependent in its pathophysiology [162]. It would be beneficial to establish the site(s) of the biothesiometer readings obtained from those in the peripheral neuropathy group of Jensen et al.’s study; unfortunately, the author was not contactable. If lower limb sensory neuropathy in one or both limbs abrogated the benefit of RIPC applied to an upper limb without any detectable sensory deficits, this would raise yet further questions about the complex mechanisms of RIPC. It could also raise the possibility that the cardioprotective effects of RIPC may have been reduced by coexisting neuropathy at other sites. Tentolouris et al. found that peripheral neuropathy and autonomic neuropathy, specifically cardiovascular autonomic neuropathy (CAN), coexisted in 45 % of cases of T2DM [163].

Peripheral neuropathy is prevalent in the T2DM population, with distal symmetric polyneuropathy being the most common [162]. Diabetic sensorimotor polyneuropathy (DSPN) is initially subclinical, and progresses to overt clinical symptoms and increased severity [164]. Research has shown that diabetic neuropathy may often be present when T2DM is first diagnosed. A study of 39 patients at diagnosis of T2DM found 82 % of the patients had electrophysiological abnormalities in peripheral nerve conduction studies, 62 % of these in more than one parameter [165].

Diabetic sensory neuropathy, particularly in relation to nociceptive fibres, decreases the release and homing of bone marrow-derived haematopoietic stem cells [141]. Evidence suggests that this stem cell response is important for angiogenesis and vascular regeneration [140, 166], including wound healing in diabetic model mice [167], and could be part of the mechanism for the late window of protection in RIPC [80, 82]. The pathogenesis of diabetic stem cell mobilopathy is also likely to involve diabetic autonomic neuropathy [168–170]. Bone marrow receives sympathetic nervous system innervation, involved in the mobilisation of stem cells [170, 171], and diabetes can lead to autonomic neuropathy of the bone marrow [168, 170].

#### Autonomic neuropathy

The autonomic nervous system (ANS) is commonly abnormal in patients with longstanding diabetes. Human and animal studies referred to above have demonstrated the integral role of the ANS in RIPC. Despite the fact that the ANS and the vagus nerve have been identified as being critical for the cardioprotection of RIPC, we are not aware of any studies published exploring the impact of autonomic neuropathy upon the effectiveness of RIPC in a clinical or human context. Decreased parasympathetic tone and dysfunction is mentioned, however, by Mastitskaya et al. as being a possible explanation for disappointing results in some studies, when their research team discovered that sectioning the posterior gastric branch of the vagus nerve in rats abrogated the effects of RIPC [72].

Diabetic autonomic neuropathy (DAN) can occur subclinically in T2DM within a year of onset, although symptoms typically develop after years [172]. The lengthy vagus nerve can be one of the first affected by neuropathy [172, 173]. DAN may occur before diabetic neuropathy involves the peripheral sensory nervous system. The commonest form of DAN is CAN, defined as impaired autonomic control of the cardiovascular system [164]. The prevalence of DAN, using various criteria, has been reported to be between 15 and 35 % in T2DM, and is commonly undiagnosed [172]. CAN, like DAN, can develop subclinically within a year of onset of diabetes [173]. The reported prevalence of CAN in T2DM also varies widely, listed as between 20 and 70 % in one review, depending on differing diagnostic criteria and duration of diabetes [174]. DePace et al. report one-third of T2DM patients at diagnosis have CAN [175].

Given knowledge about the essential role of the vagus nerve in RIPC and the high prevalence of (often undiagnosed) CAN, we suspect that the presence of autonomic neuropathy may be an unrealised but significant contributing factor in the poorer or variable response to RIPC in diabetic (and non-diabetic) patients identified in studies, and thus a confounding variable. CAN is associated with a significantly increased risk of myocardial infarction and mortality, and an independent risk factor even in those at high cardiovascular risk [174–176]. We propose a decreased response to RIPC, and by extension, potentially even to physiological conditioning such as regular physical exercise, in patients with CAN.

Another common form of DAN in T2DM is gastrointestinal autonomic neuropathy (GAN). The most frequent form of GAN is gastroparesis, which occurs in 30–50 % of patients with longstanding T2DM [177, 178]. Mastitskaya et al.’s 2016 study indicated the importance of the posterior gastric branch of the vagus nerve in the RIPC mechanism for cardioprotection in a rat model [72]. It remains to be seen whether GAN affects this specific branch of the vagus nerve, and thus could inhibit RIPC cardioprotective pathways.

Bilateral renal denervation has been shown to abrogate RIPC cardioprotective effects, concomitantly inhibiting erythropoietin production, in a murine model [70]. Erythropoietin-neutralising antibodies also abrogated the effect of RIPC in the absence of renal denervation [70]. The renal nerve consists of efferent and afferent sympathetic fibres [179], which have been hypothesised to be abnormal in severe DAN, resulting in erythropoietin deficiency (independent of nephropathy) in these patients [180].

#### Calcitonin gene related peptide

Decreased secretion of the neurotransmitters CGRP and substance P from neurons following noxious stimuli is a consequence of diabetic neuropathy [23]. The management of painful diabetic peripheral neuropathy, and neuropathic pain in non-diabetics, has been substantially improved by the use of pregabalin and gabapentin (gabapentinoids) [162, 181, 182]. Gabapentinoids are believed to inhibit the modulation of neuronal excitability, and also decrease neurotransmitters release in the presence of inflammation, including CGRP and substance P [183, 184]. This prevents nociceptive signal transmission [184].

Calcitonin gene related peptide has been identified as important in the cardioprotective neurohumoral pathways of RIPC [63], and in local pre- and post-conditioning [185]. As discussed earlier, activation of nociceptive-inducing pathways (C fibres, or capsaicin-sensitive) is crucial in the neural mechanisms of RIPC [59] as well as for the release of haematopoietic stem cells [141]. If gabapentinoids block these pathways, they potentially could reduce the effectiveness of RIPC and local ischaemic conditioning. Further well-designed studies are therefore required to clarify if anti-nociceptive pharmacotherapy may have an impact on different forms of ischaemic conditioning.

### Implications of abnormal neural pathways

We were unable to identify any articles or studies undertaken to date primarily addressing DAN, CAN, or DSPN in RIC (other than the study by Jensen et al. [64]) and their potential impact upon RIC efficacy. Indeed, despite Jensen et al.’s study findings, the presence, absence or degree of peripheral sensory neuropathy is rarely mentioned when comparing groups within a RIPC study, even when such a study is specifically investigating diabetes, or diabetes as a co-variate. As both DSPN and CAN are commonly undiagnosed and may be subclinical, yet prevalent in T2DM and even early T2DM (for CAN in particular), they have the potential to be significant but overlooked confounding variables in many studies of RIPC in diabetic participants to date, and further studies are warranted. Examination is not difficult to perform for CAN or DSPN; the screening is straightforward, non-invasive, safe, inexpensive and can be undertaken in an office setting [173, 186, 187].

## Repeat remote ischaemic conditioning

A more recent method of ischaemic conditioning is repeat ischaemic conditioning, typically administered remotely. Although often termed repeat, regular or intermittent remote ischaemic *pre*conditioning, intervention studies utilising this method are generally applied to a chronic condition (such as heart failure [188], endothelial dysfunction [189], stroke recurrence [43] or chronic wounds [44]) or following an acute ischaemic episode (such as in a lower limb [190], post-myocardial infarction [191] or cerebrovascular accident [43]). Depending on the context, the chronicity of pathological ischaemia in the cohort, and endpoints studied, this would more accurately represent combinations of pre, per and post-conditioning, reflected in the term *repeat remote ischaemic conditioning* (RRIC). Neural plasticity effects of RRIC have also been examined in an intriguing small study of healthy adult participants, demonstrating that RRIC aided motor learning and retention [192]. Although few in number and with a broad variety of endpoints, it would appear that both preclinical and clinical RRIC studies cited above have more consistently yielded significant beneficial results, particularly in comparison to clinical single-dose RIC and local ischaemic conditioning studies. Importantly, early evidence shows efficacy of RRIC in humans is not lost with advanced age. A study of 58 patients aged 80–95 years found twice daily RRIC for 180 days was effective in preventing stroke recurrence compared to sham control, without any safety concerns [193]. In contrast, RIC studies examining the impact of ageing, most assessing cardioprotection in animals, have revealed that efficacy predominantly declines with increasing age [48].

### Mechanisms

The mechanisms of RRIC are less well studied than in RIC [57]. Although it would seem logical that they may be similar to RIC, gene expression studies by Depre et al. and Shen et al. demonstrated this is not correct [194, 195]. Further, unlike the late phase of protection in RIPC, RRIC pathways do not appear to involve cyclooxygenase-2 [196]. A 4 week RRIC study by Yamaguchi et al. assessing cardiac remodelling in rats following myocardial infarction, revealed beneficial effects were associated with higher levels of miR-29a expression [188]. Kimura et al. also conducted a 4 week study, assessing endothelial function in healthy humans, and found RRIC was associated with improved endothelial function, and raised EPC, VEGF and NO levels [197]. Neutrophil function changes (including decreased adhesion and suppressed phagocytosis) and cytokine changes, associated with modulation of the systemic inflammatory response, have been identified in a 10 day human RRIC study by Shimizu et al. [85]. These changes were significant, and felt to be beneficial by the research team since tissue damage in ischaemia–reperfusion injury is facilitated by neutrophil adhesion [198]. RRIC improves skin perfusion in addition to endothelial function, even 8 days beyond RRIC intervention cessation, as demonstrated by Jones et al. [45]. It is also suspected that the effects from repeated ischaemia may be due in part to shear stress [57], leading to the mobilisation of EPCs recorded by Kimura et al. and thus neovascularisation and improved vascular function [197].

## Effectiveness of repeat remote ischaemic conditioning in type 2 diabetes

As we have demonstrated above, many factors potentially limit the benefits from ischaemic conditioning and RIC in diabetic patients. It is therefore unexpected that a recent study showed diabetic patients with refractory lower leg ulcers had significant improvement following repeat RIC [44]. In Shaked et al.’s double-blinded randomised study with sham control, 22 patients received 3 fortnightly cycles of RRIC to both arms, in addition to standard wound care. At the completion of the 6 week study, 9/22 (41 %) of the study group had complete healing of their ulcer compared to 0/12 of the control group [44]. Questionably, only one participant in each group was reported as having “neuropathy”, even though this was not listed in the exclusion criteria for the study. 46 % of the study group at commencement were documented as having local infection in the ulcer and 69 % in the control group. There were some further limitations with the study due to other important variables being unmatched between groups.

Despite the weaknesses and limitations of Shaked et al.'s small study, the wound healing rate of 41 % of refractory ulcers in just 6 weeks in a cohort of patients with very poorly controlled diabetes, after a mere 3 fortnightly remote ischaemic conditioning cycles, is not to be dismissed. Further, 14/22 patients in the intervention group reached 75 % healing at 6 weeks compared with 3/12 in the control group. 55 % of patients in the study group with ulcer infection at enrolment had completely healed wounds, compared to none in the control group with infection [44]. The findings could indicate the potential for RRIC providing significant benefit in this group, which is easily and cost-effectively administered. Should these results be reproducible in larger trials with a more satisfactorily matched control group, RRIC could revolutionize treatment of chronic diabetic foot ulcers. If this were the case, it may also point to the potential for RRIC to be effective in other chronic diabetic complications or causes of ulceration. Given the findings of improved endothelial function and increased NO, EPCs and VEGF levels in RRIC studies (albeit with predominantly healthy cohorts) discussed above, the effects of RRIC in diabetic nephropathy, neuropathy, coronary artery disease, peripheral arterial disease (PAD) and erectile dysfunction would be interesting to study. Endothelial progenitor cell therapy is being researched with some success as a potential treatment for stroke [199], another frequent complication of diabetes where RRIC may prove effective.

Shaked et al.’s study involved administration of remote conditioning. As local ischaemic preconditioning (IPC) appears to rely upon humoral and not neural mechanisms, this could mean local IPC may be relatively unaffected by the presence of DAN or DPSN. We propose repeat local IPC in the limb affected by the ulcer could possibly prove even more effective than remote ischaemic conditioning on this basis, assuming it is as safe and well tolerated as remote conditioning. Comparative studies between repeat remote and local conditioning interventions in diabetic patients with foot ulcers could be worth performing to test this theory. It is also interesting to consider Shaked et al.’s diabetic ulcer study had a control group where standard care did not involve significant pain or trauma, thus making co-conditioning from nociception less likely to confound the outcome.

Shaked et al.’s study excluded those with moderate-to-severe PAD, where ankle brachial index was <0.7. It is important to note that even in the absence of large vessel peripheral arterial disease, diabetic patients frequently have small vessel disease and tissue hypoxia in the lower limbs. Shaked et al. write that “peripheral neuropathy induces increased pressure points on the diabetic foot, which in turn lead to bouts of cutaneous IR injury” [44, p 194], from which RRIC appears likely to provide protection.

Diabetes suppresses angiogenesis in wound healing [200]. Injection of CD34+ cells into diabetic wounds has been found to improve diabetic ulcer wound healing and significantly enhanced wound revascularisation within 7 days [167]. In addition, serum CD34+/CD45-dim levels measured soon after the development of neuropathic diabetic ulcers have shown to be predictive of subsequent wound healing [201]. It is unfortunate EPCs/CD34+ cells and other factors such as VEGF were not measured in Shaked et al.’s study, but it is plausible that RRIC may have increased VEGF and/or NO, and thus EPCs (potentially overcoming mobilopathy), in the intervention group, consequently improving the impaired neovascularisation and wound healing. Both study cohorts were likely to have a high prevalence of CAN and (potentially undetected) DPSN. Neural mechanisms have not been identified nor studied in RRIC. Should the findings of Shaked et al. be replicated with more appropriately matched control and intervention groups, this may suggest that RRIC mechanisms are not as inhibited by DPSN as RIPC in diabetes. The issues regarding the beneficial effects of local ischaemic conditioning and RIC being decreased in the diabetic state, particularly relevant to the cardiomyocyte, may be less problematic with a different endpoint or target organ, such as wound healing. Similarly, it appears likely that the mechanisms in RRIC are significantly different to RIC, and coexisting neuropathy or co-medication may not result in such negative effects. Considering the evidence suggesting that RRIC leads to a state favouring neovascularisation, raised NO and EPCs, and improved microcirculatory function, one is tempted to postulate that RRIC may even slightly reverse the effects of diabetic neuropathy [202], or microangiopathy- and neuropathy-linked mobilopathy [23], hence simultaneously improving the response to each subsequently applied dose of RIC. Intermittent hypoxia is known to induce neuroplasticity and improve recovery of neurological function, including in motoneurons [192].

Raising levels of EPCs in diabetic patients is highly desirable; lower levels being associated with adverse cardiovascular outcomes [143, 203]. Current methods to increase EPCs include administration of granulocyte-colony stimulating factor, which has serious cardiovascular side effects and is often ineffective in diabetics [23, 169], or by culturing and re-administering EPCs [83]. RRIC would offer a safer, more cost-effective and easily-administered alternative, with automated RRIC devices already designed and manufactured for self-administration at home.

It is interesting to note that smoking [204] and hyperlipidaemia [205] are associated with poor bone marrow release of EPCs, in addition to a poorer response to RIPC [98, 206]. Statins [207], independent of effects on serum lipid levels, and sitagliptin [151, 159, 208] have cardioprotective properties in diabetes, and statins enhance cardioprotection in RIC [157, 206]. Studies have demonstrated that statins [209, 210] and sitagliptin [211] augment mobilisation of EPCs from bone marrow. Many of these facts may be interlinked, particularly in relation to the late phase of RIC and possibly RRIC. The effects of statins, smoking and anti-diabetic medication on RRIC are yet to be determined.

The potential use of ischaemic conditioning in PAD has received some attention. A crossover study by Delagarde et al. examined claudication distance in 20 patients with intermittent claudication after administering 3 intermittent cuff inflations and deflations [212]. A treadmill test was undertaken 5 min after completing both the RIPC and the control sham procedure, which were 7 days apart. Results did not show any alteration in claudication distance. It was surmised by Delagarde et al. that patients with PAD suffer from chronic ischaemia and reperfusion, and thus are already maximally conditioned. Consequently, they proposed that this may be why RIPC offered no additional benefit [212]. It is important to consider that performing a treadmill test 5 min after one cycle of RIPC would not allow for second window of protection (nor RRIC) effects to be assessed other than, to some extent, in those participants who performed the RIPC protocol first and then control 7 days later. In addition, only 25 % of the participants in the study were recorded as being diabetic, and presence of neuropathy was not documented. Karakoyun et al. recently undertook a key study of leg ischaemia, with a comparison between repeat local ischaemic conditioning, repeat remote ischaemic conditioning, no conditioning (ischaemic group) and sham control groups in non-diabetic rats [190]. The right iliac artery and vein were ligated to create critical limb ischaemia in all except the sham group. In the repeat local ischaemic conditioning group, the ischaemic stimulus was applied to the right leg and in the RRIC group, to the left leg. Both the RRIC and repeat local ischaemic conditioning group received 3 cycles of 10 min of ischaemia followed by 10 minutes of reperfusion daily until sacrificed at days 1,7,14 and 30. The ischaemic group and sham group received no further intervention. The findings of Karakoyun et al.’s study were that RRIC resulted in increased angiogenesis in the ischaemic right limb, although with no improvement in blood flow. The repeat local ischaemic conditioning group demonstrated both improved angiogenesis and blood flow. These positive findings were significant when compared to the ischaemic and sham control groups. EPCs were also measured, and were particularly higher in the RRIC group [190].

## Potential concerns for repeat remote ischaemic conditioning in type 2 diabetes

The optimum dose and limb/s used for RRIC are not known [56, 87, 213]; considerable variation in study protocols exists without losing beneficial effects [57]. It is unknown if hyperconditioning may be problematic [213]. No adverse effects from administration of either RIC [95] or RRIC, other than transient minor bruising, have been reported, even with frequent and extended administration of RRIC to two limbs concurrently in a cohort with known vascular disease [43].

Despite RIC and RRIC being reported as very safe and well tolerated in virtually all studies to date [87], a concern of ours, shared by Heyman et al. is the potential for worsening or increased risk of proliferative diabetic retinopathy [214]. Diabetic foot ulcers occur particularly in patients with longstanding and/or poorly controlled diabetes, which are in turn both highly significant risk factors for diabetic retinopathy [215]. VEGF is a key factor in diabetic retinopathy progression [216]. Kimura et al. found VEGF levels to be increased after one month of 6 times daily RRIC [197]. In contrast, Czeiger et al. did not identify raised VEGF levels, despite increased EPC numbers following RIPC [83]. Further, increased EPC levels are associated with a decreased incidence of diabetic complications, including retinopathy [142, 143, 170, 217]. We recommend that future studies of ischaemic conditioning in diabetes should assess VEGF and EPC levels, particularly if studying repeat ischaemic conditioning over long periods in diabetic patients. It is possible that the presence or high risk of diabetic retinopathy may be found to be a future contraindication to RRIC, although we are not aware of any evidence proving such a link. Further information from studies is required to establish the relationship between frequency, dose and form of ischaemic conditioning, VEGF and EPC levels, and any progression of diabetic retinopathy, to guide recommendations.

## Conclusions

The mechanisms associated with different forms of ischaemic conditioning have been discussed in the context of T2DM and diabetic lower extremity ulceration. Neural pathways may frequently be affected in diabetic cohorts with known or undiagnosed peripheral sensory neuropathy, CAN and/or GAN. Ischaemic conditioning studies performed on diabetic patients have shown varying and mixed results. Conclusions drawn from earlier studies with diabetic patients should be re-evaluated carefully, particularly in research protocols where such neuropathies were not screened for, disclosed or matched between study and control groups. We feel detailed investigation is required to assess the impact of diabetic neuropathies upon RIPC and repeat RIC in particular. Although so far there have been no reported concerns with safety in studies, further research and consideration are required to assess the levels of VEGF, and all alterations of humoral effectors as they become known in RRIC, and establish that these are not harmful in the longer term. Repeat RIC may be an effective new method to consider in the treatment of diabetic lower extremity ulceration and peripheral arterial disease that is inexpensive, easy to administer in any location, and well tolerated.

## Abbreviations

T2DM: type 2 diabetes mellitus
RIC: remote ischaemic conditioning
RIPC: remote ischaemic preconditioning
RRIC: repeat remote ischaemic conditioning
ANS: autonomic nervous system
DSPN: diabetic sensorimotor polyneuropathy
DAN: diabetic autonomic neuropathy
CAN: cardiac autonomic neuropathy
GAN: gastrointestinal autonomic neuropathy
EPCs: endothelial progenitor cells
VEGF: vascular endothelial growth factor
CGRP: calcitonin gene related peptide
NO: nitric oxide
CABG: coronary artery bypass graft
PCI: percutaneous coronary intervention
AMI: acute myocardial infarction
PAD: peripheral arterial disease
DPP-4: Dipeptidyl peptidase-4
